# Increased age at first-mating interacting with herd size or herd productivity decreases longevity and lifetime reproductive efficiency of sows in breeding herds

**DOI:** 10.1186/s40813-019-0142-9

**Published:** 2020-02-06

**Authors:** Yuzo Koketsu, Ryosuke Iida, Carlos Piñeiro

**Affiliations:** 1 School of Agriculture, Meiji University, Higashi-mita 1-1-1, Tama-ku, Kawasaki, Kanagawa 214-8571 Japan; 2 PigCHAMP Pro Europa S.L, c/Calle Dámaso Alonso, 14, 40006 Segovia, Spain

**Keywords:** Farm management, Lifetime performance, Herd-life days, sow life days

## Abstract

**Background:**

Our objectives were to characterize sow life and herd-life performance and examine two-way interactions between age at first-mating (AFM) and either herd size or herd productivity groups for the performance of sows. Data contained 146,140 sows in 143 Spanish herds. Sow life days is defined as the number of days from birth to removal, whereas the herd-life days is from AFM date to removal date. Herds were categorized into two herd size groups and two productivity groups based on the respective 75th percentiles of farm means of herd size and the number of piglets weaned per sows per year: large (> 1017 sows) or small-to-mid herds (< 1017 sows), and high productivity (> 26.5 piglets) or ordinary herds (< 26.5 piglets). A two-level liner mixed-effects model was applied to examine AFM, herd size groups, productivity groups and their interactions for sow life or herd-life performance.

**Results:**

No differences were found between either herd size or herd productivity groups for AFM or the number of parity at removal. However, late AFM was associated with decreased removal parity, herd-life days, herd-life piglets born alive and herd-life annualized piglets weaned, as well as with increased sow life days and herd-life nonproductive days (*P* < 0.05). Also, significant two-way interactions between AFM and both herd size and productivity groups were found for longevity, prolificacy, fertility and reproductive efficiency of sows. For example, as AFM increased from 190 to 370 days, sows in large herds decreased herd-life days by 156 days, whereas for sows in small-to-mid herds the decrease was only 42 days. Also, for the same AFM increase, sows in large herds had 5 fewer sow life annualized piglets weaned, whereas for sows in small-to-mid herds this sow reproductive efficiency measure was only decreased by 3.5 piglets. Additionally, for ordinary herds, sows in large herds had more herd-life annualized piglets weaned than those in small-to-mid herds (*P* < 0.05), but no such association was found for high productivity herds (*P* > 0.10).

**Conclusion:**

We recommend decreasing the number of late AFM sows in the herd and also recommend improving longevity and lifetime efficiency of individual sows.

## Background

Lifetime performance of sows can be measured as longevity, prolificacy, fertility and reproductive efficiency. Longevity is commonly measured as the number of parity at removal [1], sow herd-life days [2, 3] or sow life days which are from the birth date to the removal date. Using sow life days or herd-life days as a denominator, annualized piglets weaned can be used as an integrated measurement for reproductive efficiency of individual sows combining fertility, prolificacy and longevity. Prolificacy is the herd-life numbers of piglets born alive, whereas fertility can be represented by herd-life nonproductive days which includes weaning-to-first-mating interval and re-service interval.

A common benchmarking measurement to monitor reproductive efficiency within the herd or to compare productivity of different herds is the number of pigs weaned per sow per year (PSY) [1, 4]. However, higher PSY is not directly associated with higher longevity measured as the mean parity of removed sows in ordinary conditions [5]. So, culling a sow at low parity does not necessarily decrease reproductive efficiency measured as PSY. In fact, some producers cull low parity sows with fewer piglets born alive to maintain high herd productivity [6, 7]. However, sows that are culled at low parity are not able to realize their latent potential life days. Also, high sow longevity can increase the profit per sow because lifetime piglets weaned by parity 3 or higher sows retrieve the initial cost of a replacement gilt [8].

Age of gilts at first-mating (AFM), which is commonly recorded by producers, is a measurement to predict sow reproductive performance [9], although recording age at first estrus and heat-no-service events are recommended as better measures [10]. Also, herd size and PWSY are herd-level factors for sow reproductive performance [9, 11]. However, there are no reports on interactions between such herd-level factors and AFM for sow life and herd-life reproductive performance. Therefore, the objectives of the present study were to characterize sow life days or herd-life days, prolificacy, fertility and reproductive efficiency of sows in breeding herds and examine two-way interactions between AFM and these two herd-level factors for sow lifetime performance.

## Methods

### Studied herds

A veterinary consultancy firm (PigCHAMP pro Europa S.L., Segovia, Spain) requested all client producers to mail their PigCHAMP data files on a regular basis to build up a sow database for their veterinary services. In July 2017, sow life and herd-life reproductive performance records of sows in 155 Spanish herds, which allowed their data to be used for research, were extracted from the database. However, 12 of the 155 herds were excluded from the study because they had no birthdates recorded.

In the present study, mean herd size (± SEM) in the remaining 143 herds was 856 ± 61 sows with a range from 87 to 3669 sows between 2011 and 2016. Also, the herd mean of PSY (± SEM) in the studied herds was 25.8 ± 0.24 ranging between 11.6 and 33.3. Sows in the studied herds were mainly crossbreds between Landrace and Large White, and replacement gilts were either purchased from international breeding companies or home-produced through internal multiplication programs with sire and dam lines purchased from breeding companies.

### Study design, data and exclusion criteria

This observational study mimicked a two by two factorial arrangement design, using the main effects of two herd size groups and two herd productivity groups. Data included sow life and herd-life performance records of 152,412 sows which were entered the herds during 2011–2013, and were removed by December 2016. Sow records were excluded if sows’ AFMs were 159 days or less, or 401 days or more (5218 sows) because first mating at such an early or advanced age was considered extreme. Hence, the final dataset contained sow life and herd-life performance records of 147,194 sows.

### Categories and definitions

Herds were categorized into two by two groups based on the 75th percentiles of the farm means of herd size and PSY during 2011–2016: large herds (> 1017 sows) or small-to-mid herds (< 1017 sows), and high productivity herds (> 26.5 piglets) or ordinary herds (< 26.5 piglets). The 75th percentile was chosen so that each of the four sow groups contained at least 10% of the sows. Also, to examine frequency distributions (%), sows were categorized into eight 30-day AFM interval groups between 160 and 400 days.

In this manuscript, lifetime means both sow life days and herd-life days. Sow life days are the number of days from birth to removal, whereas herd-life days are the number of days from day of gilt first-mating (AFM date) to removal date. Also, sow life and herd-life annualized piglets weaned are respectively the total number of pigs weaned during the sow’s life divided by sow life days, and herd-life days × 365.25. The number of parity at removal includes both culled sows and dead sows.

### Statistical analysis

All analyses were conducted using SAS University Edition (SAS Inst. Inc., Cary, NC). Also, the data was subjected to a two-level liner mixed-effects model using the MIXED procedure to examine AFM as a continuous variable, the quadratic expressions of AFM, the two herd size groups, the two productivity groups and the two-way interactions for lifetime performance of sows. The AFM was centered at the grand mean value. Also, AFM was analyzed by the two herd size groups, the two productivity groups and the two-way interaction. Levels 1 and 2 were a sow and a herd, respectively, to account for the clustering of sows within a herd (random statement). The following factors were also included as fixed effects for all the models: quarterly season of herd entry, entry year and the entry seasons within the entry year. Quarterly seasons were January–March, April–June, July–September and October–December. For all analyses, the significance level was set at 0.05.

The intraclass correlation coefficients (ICC) were calculated by the following equation [12] to assess the variation in the performance examined that could be explained by the herd: ICC (individual records within the same herd) = ${\sigma}_v^2/\left({\sigma}_v^2+{\sigma}_e^2\right)$, in which ${\sigma}_v^2$ is the between-herd variance and ${\sigma}_e^2$ is the assumed variance at the individual record level.

## Results

**Fig. 1:**
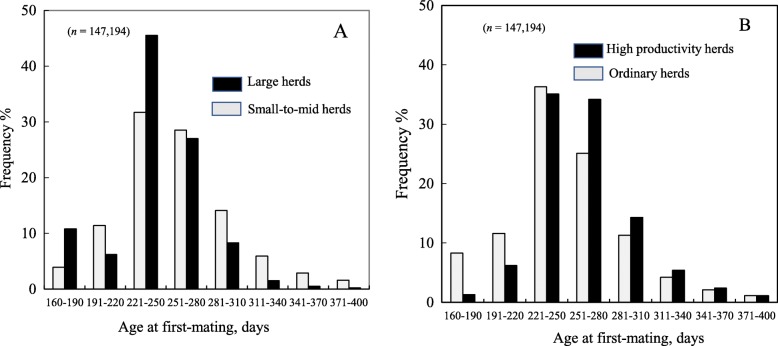
Frequency distributions (%) of gilt age at first-mating for two herd size groups (**a**) and two productivity groups (**b**)

Means of the number of parity at removal, sow life days, sow herd-life days, herd-life piglets born alive, herd-life annualized piglets weaned and sow life annualized piglets weaned (± SEM) were 4.90 ± 0.01, 1088 ± 0.9 days, 834 ± 0.9 days, 68.3 ± 0.09 piglets, 25.5 ± 0.02 piglets and 18.3 ± 0.02 piglets, respectively (Table 1). Also, mean AFM was 254 ± 0.11 days. Figure 1 shows the frequency distributions (%) of the eight AFM groups for the two herd size groups and two productivity groups. Over 50% of gilts in all these groups were first-mated between 221 and 280 days. Also, the large herd group had 11% of gilts first-mated at only 160–190 days of age.

**Table 1:** Lifetime performance and reproductive performance of sows in 143 herds

Measurements	*n*	Mean	SEM	Median (Interquartile range)
Age at first-mating	147,194	254	0.11	249 (234–273)
Longevity				
Number of parity at removal	147,194	4.90	0.01	5 (2–7)
Sow life days	147,194	1088	0.92	1138 (829–1346)
Herd-life days	147,194	834	0.92	882 (575–1096)
Prolificacy				
Herd-life piglets born alive	135,865	68.3	0.09	71 (42–93)
Herd-life piglets weaned	135,865	59.9	0.08	64 (38–81)
Fertility				
Herd-life non-productive days	135,865	72.0	0.15	56 (33–98)
Reproductive efficiency				
Herd-life annualized piglets weaned	135,865	25.5	0.02	25 (23–28)
Sow life annualized piglet weaned	135,865	18.3	0.02	20 (15–22)

Table 2 shows that there were no differences between herd size or between herd productivity groups for either AFM or the number of parity at removal (*P* > 0.10). Sows in large herds had 58 fewer sow life days and 58 fewer herd-life days than small-to-mid herds, but there were no significant differences in these measurements between the productivity groups (*P* > 0.10). However, high productivity groups had 5.0 more herd-life piglets weaned, 5.2 more herd-life piglets born alive and 2.1 more sow life annualized piglets weaned than ordinary herds, but there were no such differences between the herd size groups (*P* > 0.10).

**Table 2:** Comparisons of performance measurements (Means and SEM) between two herd size and between two productivity groups^1^

	Herd size groups		Herd productivity groups	
Measurements	Small-to-mid herds	Large herds	Ordinary herds	High productivity herds
n	102,528	44,666	99,680	47,514
Age at first-mating	264 ± 4	241 ± 8	241 ± 6	264 ± 7
Longevity				
Number of parity at removal	5.17 ± 0.10	4.88 ± 0.12	5.01 ± 0.09	5.03 ± 0.13
Sow life days	1056 ± 14 ^a^	998 ± 19 ^b^	1038 ± 14	1017 ± 19
Sow herd-life days	801 ± 14 ^a^	743 ± 18 ^b^	783 ± 14	762 ± 19
n	83,600	52,265	79,594	56,271
Prolificacy				
Herd-life piglets born alive	69.2 ± 1.2	66.0 ± 1.5	65.0 ± 1.2 ^b^	70.2 ± 1.6 ^a^
Herd-life piglets weaned	60.0 ± 1.0	58.0 ± 1.2	56.5 ± 0.9 ^b^	61.5 ± 1.3 ^a^
Reproductive efficiency				
Sow life annualized piglet weaned	18.8 ± 0.2	18.7 ± 0.2	17.7 ± 0.1 ^b^	19.8 ± 0.2^a^

^1^Means and SEs were estimated by the models

^a,b^Different superscripts within a row represent significant differences in means (*P* < 0.05)

No two-way interaction was found between herd size and herd productivity groups for AFM or any lifetime performance (*P* > 0.10) except for herd-life annualized pigs weaned and nonproductive days (Table 3). The significant association for herd-life annualized piglets weaned only occurred with ordinary herds, where sows in large herds had more herd-life annualized piglets weaned than those in small-to-mid herds (*P* < 0.05); no such association was found for high productivity herds (*P* > 0.10).

**Table 3:** Comparisons of performance measurements (Means and SEM) between two herd size and two productivity groups^1^

	Productivity groups	
Herd size groups	Ordinary herds	High productivity herds
n		
Small-to-mid farms	37,743	14,522
Large farms	41,851	41,749
Herd-life annualized piglets weaned, pigs		
Small-to-mid farms	23.5 ± 0.2 ^b,y^	27.1 ± 0.3 ^a^
Large farms	24.5 ± 0.3 ^b,x^	26.9 ± 0.3 ^a^
Herd-life nonproductive days		
Small-to-mid farms	95.0 ± 2.3 ^a,x^	66.9 ± 4.7 ^b^
Large farms	77.1 ± 4.6 ^y^	67.2 ± 5.1

^1^Means and SEs were estimated by the models

^a,b^ Different superscripts within a row represent significant differences in means (*P* < 0.05)

^x,y^ Different superscripts within a column represent significant differences in means (*P* < 0.05)

**Fig. 2:**
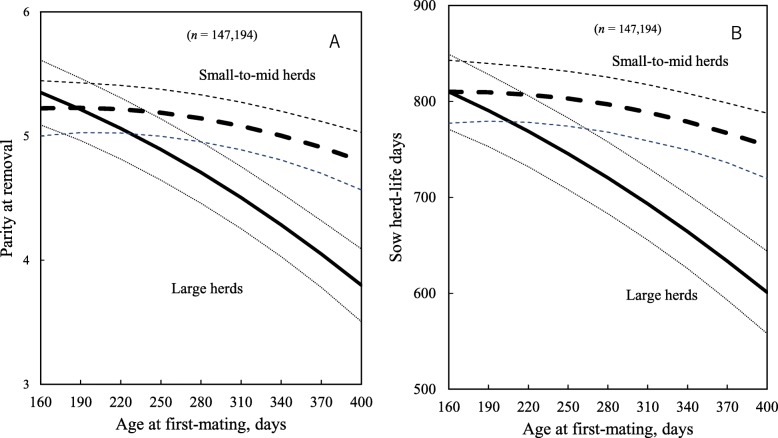
Predicted number of parity at removal (**a**) and sow herd-life days (**b**) at different gilt ages at first-mating. Dotted lines show 95% confidence intervals

**Fig. 3:**
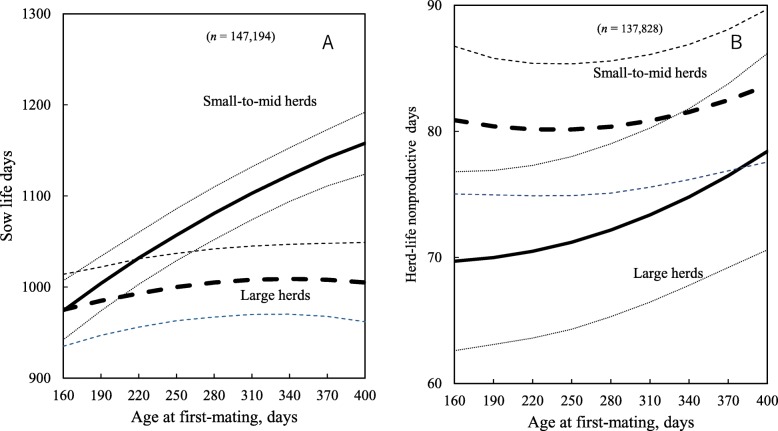
Predicted sow life days (**a**) and herd-life nonproductive days (**b**) at different gilt ages at first-mating. Dotted lines show 95% confidence intervals

Late AFM was associated with decreased sow longevity, measured as the number of parity at removal or herd-life days, as well as with prolificacy, fertility and sow reproductive efficiency measures (Tables 4 and 5; *P* < 0.05). Also, a significant two-way interaction between AFM and herd size groups was found for longevity, prolificacy, fertility and reproductive efficiency of sows (Tables 3 and 4; *P* < 0.05). For example, as AFM increased from 190 to 370 days, the parity at removal for sows in large herds decreased by 1.2, whereas for sows in small herds it decreased by only 0.3 (Fig. 2a). Also, for the same increase in AFM, the herd-life days of sows in large herds decreased by 157 days, whereas it only decreased by 42 days for sows in small herds (Fig. 2b). In contrast, the sow life days of sows in small-to-mid herds increased by 138 days, over the same AFM range, whereas it only increased by 23 days for sows in large herds (Fig. 3a).

**Table 4:** Estimates of fixed factors and random effect variance in the models for longevity and fertility measurements

	Sow life days	Herd-life days	Herd-life nonproductive days	Parity at removal
Fixed and random effects	Estimate (± SE)	Estimate (± SE)	Estimate (± SE)	Estimate (± SE)
Intercept	936.64 (28.19)	682.21 (28.19)	61.04 (5.1580)	4.49 (0.18)
Age at first-mating	0.2381 (0.0005)*	− 0.7619 (0.0596)*	0.0172(0.0099)*	−0.0055 (0.0004)*
Age at first-mating squared	− 0.0010 (0.0005)*	− 0.0010 (0.0005)*	− 0.00013 (0.00009)	− 0.000009 (0.000004)*
Herd size groups				
Small herds	25.8010 (38.2240)*	25.8010 (38.2240)*	−0.2871 (6.9943)*	0.1568 (0.2527)
Age at first-mating x Herd size				
Age at first-mating x Small-to-mid herds	0.6360 (0.0710)*	0.6360 (0.0710)*	−0.0246 (0.0119)*	0.0047 (0.0005)*
Herd productivity groups				
Ordinary herds	−10.4434 (37.7297)	−10.4434 (37.7297)	9.8396 (6.9064)*	−0.1535 (0.2493)
Age at first-mating x Herd productivity				
Age at first-mating x Low herds	−0.0949 (0.0704)	−0.09492(0.0704)	0.0219 (0.0117)	−0.0008 (0.0005)
Herd size x Herd productivity				
Small-to-mid herds x Ordinary herds	62.9712 (47.4994)	62.9712 (47.4994)	18.4513 (8.6888)*	0.2775 (0.3141)
Intercept variance at herd level	9372	9372	298	0.40
ICC (records within the same herd), %	6.3	6.3	7.6	5.3

*indicates *P* < 0.05

**Table 5:** Estimates of fixed factors and random effect variance in the models for reproductive efficiency and prolificacy measurements

	Sow life annualized piglets weaned	Herd-life annualized piglets weaned	Lifetime piglets weaned	Lifetime piglets born alive
Fixed and random effects	Estimate (± SE)	Estimate (± SE)	Estimate (± SE)	Estimate (± SE)
Intercept	19.7364 (02965)	27.6631 (03307)	57.4532 (1.8624)	64.1534 (4.1551)
Age at first-mating	- 0.0295 (0.0008)*	−0.0054 (0.0008)*	−0.0529 (0.0046)*	−0.0548(0.0054)*
Age at first-mating squared	0.000009 (0.000007)	−0.0001 (0.000007)	−0.00004 (0.00005)	− 0.00001 (0.00005)
Herd size groups				
Small herds	0.3781 (0.4014)	0.2285 (0.4481)	2.0886 (2.5230)	0.0779 (0.0087)
Age at first-mating x Herd size				
Age at first-mating x Small-to-mid herds	0.0076 (0.0010)*	0.0023 (0.0009)*	0.0402 (0.0055)*	0.0469 (0.0154)*
Herd productivity groups				
Ordinary herds	−1.7451 (0.3955)*	−2.3670 (0.4421)*	−4.9031 (2.4883)	−5.0191 (5.8705)
Age at first-mating x Herd productivity				
Age at first-mating x Ordinary herds	0.0036 (0.0010)*	0.0023 (0.0009)*	0.0012 (0.0051)	−0.03710 (0.0068)
Herd size x Herd productivity				
Small-to-mid herds x Ordinary herds	−0.6814 (0.4994)	−1.2828 (0.5571)*	−0.1372 (3.1372)	− 0.4006 (3.9637)
Intercept variance at herd level	1.03	1.34	42.0	67.5
ICC (records within the same herd), %	4.0	4.4	5.1	5.8

*indicates *P* < 0.05

**Fig. 4:**
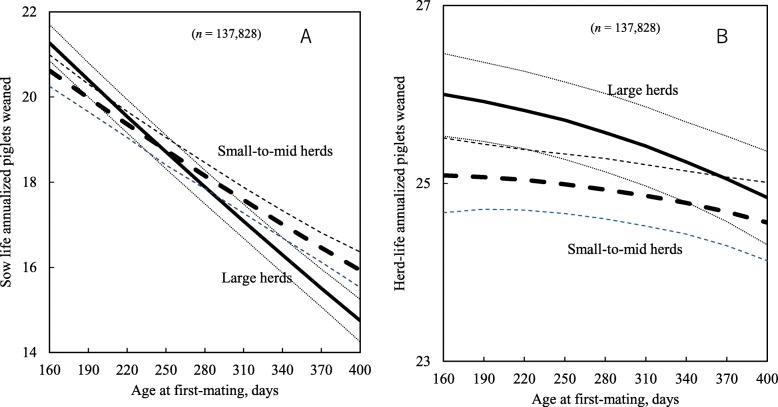
Predicted sow life annualized piglets weaned (**a**) and herd-life annualized piglets weaned (**b**) at different age at first-mating. Dotted lines show 95% confidence intervals

With regard to fertility and efficiency, as AFM increased from 190 to 370 days, herd-life nonproductive days for sows in large herds decreased by 6.5 days, whereas it decreased by only 2.1 days for sows in small-to-mid herds (Fig. 3b). Also, for the same increase in AFM, herd-life annualized piglets weaned for sows in large herds decreased by 1 piglet, whereas it decreased by only 0.4 piglets for sows in small-to-mid herds (Fig. 4a and b).

**Fig. 5:**
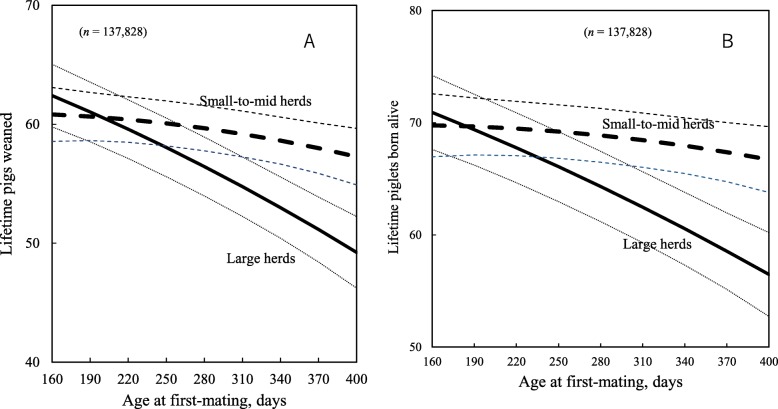
Predicted herd-life piglets weaned (**a**) and herd-life piglets born alive (**b**) at different age at first-mating. Dotted lines show 95% confidence intervals

In addition, as AFM increased from 190 to 370 days, sow life annualized piglets weaned for sows in large herds decreased by 5 piglets, compared with only 3.5 piglets for sows in small-to-mid herds. Furthermore, when AFM increased from 190 to 370 days, herd-life piglets weaned and piglets born alive for sows in large herds decreased by 10 and 11 piglets, respectively, but only fell by 2.7 and 2.3 piglets, respectively, for sows in small-to-mid herds (Fig. 5a and b).

**Fig. 6:**
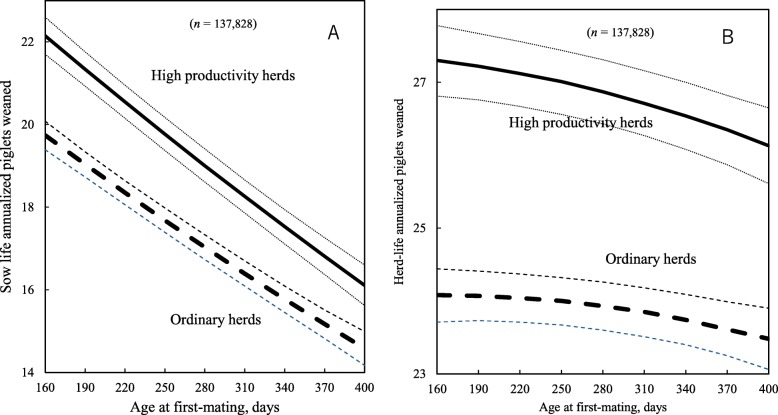
Predicted sow life annualized piglets weaned (**a**) and herd-life annualized piglets weaned (**b**) at different age at first-mating. Dotted lines show 95% confidence intervals

Additionally, a two-way interaction between AFM and herd productivity groups was found significant for sow life and herd-life annualized piglets weaned (Table 3; *P* < 0.05). As AFM increased from 190 to 370 days, sows in high productivity herds had 4.5 fewer sow life annualized piglets weaned and 1 fewer herd-life annualized piglet weaned, whereas for sows in ordinary herds the respective decreases were 4 and 0.5 piglets (Fig. 6a and b).

## Discussion

The means of sow herd-life days and number of parity at culling in the studied herds were 834 herd-life days and 4.9, respectively, which are higher than equivalent values of 467 herd-life days and removal at parities 3–4 reported in U.S.A. studies [2, 13]. Therefore, it suggests that the studied Spanish herds have higher sow longevity than typical U.S.A. herds. The U.S.A. herds appear to have different culling policies from European herds. Meanwhile, a European study reported 735 herd-life days and 4.4 parities at culling which are similar to this study’s values [14].

The differences in this study between lifetime performance measurements for herd size groups and productivity groups indicate that herd size affects sow longevity, whereas productivity is related to prolificacy, fertility and sow reproductive efficiency. So it is possible that large herds implement more strict culling policies than small-to-mid herds, whereas high productivity herds have better sow reproductive efficiency than ordinary herds. Also, it could be that care of late AFM sows in large herds may not be good as in small herds. It is well known that high productivity herds, based on PSY, have higher farrowing rates and lower repeat rates than ordinary herds, in order to reduce nonproductive days [15, 16].

Large herds administrated by large corporations could have more human resources, advanced facilities and technologies than small herds [17]. However, the fact that herd size only affected herd-life annualized piglets weaned in ordinary herds, and not in high productivity herds, indicates that herd size is only important for reproductive efficiency in ordinary herds, not in high productivity herds. So, small-to-mid herds can still compete with large herds in terms of improving sow reproductive efficiency. Also, the present study found no interaction between herd size and productivity groups for longevity or prolificacy, which indicates that, regardless of herd size or herd PSY, producers can improve longevity and prolificacy of sows.

The AFM currently recommended is to start mating gilts from 203 days of age in order to let replacement gilts have enough time to develop sufficient body weight and body fat needed for pregnancy [18]. The mean AFM and distributions in the present study are similar to published results in other countries [19–21]. Some large herds had early AFM of 160–190 days, but such early AFM is not recommended because gilts with early AFM may not have sufficient body fat, body weight and maturity of genital organs [10, 18, 19]. It is possible that these farms might have tried to meet their target number of mated sows during a certain period by mating young replacement gilts [9].

This study clearly showed that sows with late AFM were associated with decreased longevity, prolificacy, fertility and reproductive efficiency measurements except for sow life days. However, AFM can be advanced to some degree by a puberty stimulation program for gilts [10, 22], although innately fertile sows do tend to have early AFM and high lifetime performance [22]. Additionally, it is recommended that producers record and pay close attention to gilt age at first-estrus, heat-no-service events and AFM [22].

Our study also revealed that the decrease in the number of parity at removal and sow herd-life days was greater for large herds than for small-to-mid herds as AFM increased. Furthermore, large herds increased sow life days by only 0.13 days per day of AFM, whereas small-to-mid herds increased sow life days by 0.77 days.

Sows in large herds with AFM 280 days or later had fewer piglets born and piglets weaned in their herd life than equivalent sows in small-to-mid herds. It suggests that large herds were more likely to cull sows with late AFM than small-to-mid herds, because both the numbers of piglets born and piglets weaned in sows’ herd life are negatively associated with those sows’ parity at culling [1, 9].

Increasing AFM decreased herd-life nonproductive days more in large herds than in small-to-mid herds. The reason is probably that large herds are more concerned about prolonged nonproductive days in late AFM gilts than small-to-mid herds. Decreasing nonproductive days is critical to improve sow reproductive efficiency [1, 9]. It is well known that late AFM sows have more piglets born alive in parity 1 [19, 23] but have a greater weaning-to-first-mating interval, and consequently prolonged nonproductive days or lower fertility than early AFM sows [21, 24]. Also, late AFM gilts are more likely to have late returns than early AFM gilts [25], and late return gilts are thought to have low corpora lutea functions, and low progesterone concentrations [26]. So, late AFM gilts are likely to become low-efficiency sows by having increased nonproductive days due to reproductive failure and prolonged culling intervals [19, 27]. Also, low performance in late AFM sows is thought to be related to being overweight at mating [21] and low longevity.

However, as AFM increased, efficiency measurements, such as the number of annualized piglets weaned, decreased more steeply in large herds than in small-to-mid herds. Again, large herds might have put too much culling pressure on late AFM sows in terms of the efficiency. Culling low parity sows with fewer piglets produced and replacing them with replacement gilts does not recover the sow life days or herd-life days [2]. In other words, it is not possible for weaned piglets of sows culled at parity 1 or 2 to offset the culled mother sow life days (e.g. 300 or more sow life days). In fact, the lifetime net income or net present value of a sow becomes negative when it is culled at parity 1–3 [8, 28]. Alternatively, large herds might have purchased a replacement gilt package from breeding companies on the basis of herd PSY, not sow longevity or individual sow efficiency.

As AFM increased, both high productivity and ordinary herds decreased sow life annualized piglets weaned more steeply than herd-life annualized piglets weaned. This indicates that sow life annualized piglets weaned is more sensitive to increasing AFM and decreasing longevity than herd-life annualized piglets weaned.

There was an interaction between herd size and AFM for all the performance measures we examined. It appears that herd size was an important factor for the longevity, prolificacy, fertile and reproductive efficiency of the sows in our studied herds in Spain, which is famous for swine production managed by large corporations [29].

## Conclusion

Good measures for monitoring and improving lifetime performance of sows are sow life days and herd-life days, for longevity, and annualized piglets weaned for reproductive efficiency. It is also important for producers to record AFM with age at first estrus. Regardless of herd size, we recommend decreasing the number of late AFM sows in the herd by a puberty stimulating program. In particular, large herds should reconsider their culling policy and care taking for late AFM sows, because late AFM negatively affected sows’ longevity, prolificacy, fertility and reproductive efficiency more in large herds than in small-to-mid herds. Finally, we recommend improving longevity and lifetime reproductive efficiency of individual sows in breeding herds.

## Data Availability

The dataset analyzed during the current study is not publicly available because producers’ privacy could be compromised.

## Abbreviations

AFC: Age at first-mating
ICC: Intraclass correlation coefficients

## References

[1] Dial GD, Marsh WE, Polson DD, Vaillancourt JP, Leman AL, Straw BE, Mengeling WL, D’Allaire S, Taylor DJ (1992). Reproductive failure: differential diagnosis. Disease of swine.

[2] Rodriguez-Zas SL, Southey BR, Knox RV, Connor JF, Lowe JF, Roskamp BJ (2003). Bioeconomic evaluation of sow longevity and profitability. J Anim Sci.

[3] Sasaki Y, Koketsu Y (2008). Mortality, death interval, survivals, and herd risk factors for female pigs in commercial breeding herds. J Anim Sci.

[4] PigCHAMP. PigCHAMP benchmarking. http://www.pigchamp.com/ benchmarking/benchmarking-summaries. Accessed 30 Nov 2019.

[5] Koketsu Y (2007). Longevity and efficiency associated with age structures of female pigs and herd management in commercial breeding herds. J Anim Sci.

[6] Tani S, Piñeiro C, Koketsu Y (2018). Culling in served females and farrowed sows at consecutive parities in Spanish pig herds. Porcine Health Manag.

[7] Bergman P, Gröhn YT, Rajala-Schultz P, Virtala A-M, Oliviero C, Peltoniemi O, Heinonen M (2018). Sow removal in commercial herds: patterns and animal level factors in Finland. Prev Vet Med.

[8] Stadler KJ, Lacy C, Cross TL, Glenn E, Conatser GE (2003). Financial impact of average parity of culled females in a breed-to-wean swine operation using replacement gilt net present value analysis. J Swine Health Prod.

[9] Koketsu Y, Tani S, Iida R (2017). Factors for improving reproductive performance of sows and herd productivity in commercial breeding herds. Porcine Health Manag.

[10] Patterson J, Triemert E, Gustafson B, Werner T, Holden N, Pinilla JC, Foxcroft G (2016). Validation of the use of exogenous gonadotropins (PG600) to increase the efficiency of gilt development programs without affecting lifetime productivity in the breeding herd. J Anim Sci.

[11] Bergman P, Munsterhjelm C, Virtala A-M, Peltoniemi O, Valros A, Heinonen M (2019). Structural characterization of piglet producing farms and their sow removal patterns in Finland. Porcine Health Manag.

[12] Dohoo IR, Martin SW, Stryhn H (2009). Veterinary epidemiologic research.

[13] Lucia T, Dial GD, Marsh WE (1999). Estimation of lifetime productivity of female swine. J Am Vet Med Assoc.

[14] Engblom L, Lundeheim N, Dalin AM, Andersson K (2007). Sow removal in Swedish commercial herds. Livest Sci.

[15] Koketsu Y, Sasaki Y (2009). By-parity nonproductive female days in commercial swine herds. J Vet Med Sci.

[16] Sasaki Y, Saito H, Shimomura A, Koketsu Y (2011). Consecutive reproductive performance after parity 2 and lifetime performance in sows that had reduced pigs born alive from parity 1 to 2 in Japanese commercial herds. Livest Sci.

[17] King VL, Koketsu Y, Reeves D, Xue JL, Dial GD (1998). Management factors associated with swine breeding-herd productivity in the United States. Prev Vet Med.

[18] PIC manuals: Gilt and sow management guidelines, 2017. http://www.picrsa.co.za/manuals/ accessed 31 Jul 2019.

[19] Schukken YH, Buurman J, Huirne RBM, Willemse AH, Vernooy JCM, van den Broek J, Verheijden JHM (1994). Evaluation of optimal age at first conception in gilts from data collected in commercial swine herds. J Anim Sci.

[20] Koketsu Y, Takahashi H, Akachi K (1999). 1999. Longevity, lifetime pig production, and age at first mating in a cohort of gilts over 6 years. J Vet Med Sci.

[21] Roongsitthichai A, Cheuchuchart P, Chatwijitkul S, Chantarothai O, Tummaruk P (2011). Influence of age at first estrus, body weight, and average daily gain of replacement gilts on their subsequent reproductive performance as sows. Livest Sci.

[22] Patterson J, Foxcroft G (2019). Gilt Management for Fertility and Longevity. Animals..

[23] Tummaruk P (2012). Effects of season, outdoor climate and photo period on age at first observed estrus in landrace x Yorkshire crossbred gilts in Thailand. Livest Sci.

[24] Saito H, Sasaki Y, Koketsu Y (2011). Associations between age of gilts at first mating and lifetime performance or culling risk in commercial herds. J Vet Med Sci.

[25] Tani S, Piñeiro C, Koketsu Y (2016). Recurrence patterns and factors associated with regular, irregular and late returns-to-service of female pigs and their lifetime performance on southern European farms. J Anim Sci.

[26] Bertoldo MJ, Holyoake PK, Evans G, Grupen CG (2012). Seasonal variation in the ovarian function of sows. Reprod Fert Develop.

[27] Iida R, Piñeiro C, Koketsu Y (2015). High lifetime and reproductive performance of sows in southern European Union commercial farms can be predicted by high numbers of pigs born alive at parity one. J Anim Sci.

[28] Sasaki Y, McGgartt I, Koketsu Y (2012). Assessment of lifetime economic returns of sow by parity at culling in commercial breeding herds. J Vet Epi.

[29] Rocadembosch J, Amador J, Bernaus J, Font J, Fraile LJ (2016). Production parameters and pig production cost: temporal evolution 2010–2014. Porcine Health Manag.

